# Correlations between Gustatory, Olfactory, Cognitive Function, and Age in Healthy Women

**DOI:** 10.3390/nu16111731

**Published:** 2024-05-31

**Authors:** Fabrizio Sanna, M. Paola Castelli, Rafaela Mostallino, Francesco Loy, Carla Masala

**Affiliations:** Department of Biomedical Sciences, University of Cagliari, Cittadella Universitaria, SP 8 Monserrato, 09042 Cagliari, Italy; castelli@unica.it (M.P.C.); rafaela.mostallino@unica.it (R.M.); floy@unica.it (F.L.); cmasala@unica.it (C.M.)

**Keywords:** aging, taste, olfaction, chemosensory dysfunction, cognitive impairment, women

## Abstract

Aging is a progressive physiological degeneration associated with a decline in chemosensory processes and cognitive abilities and a reduction in synaptic plasticity. The biological bases of ageing are still not completely understood, and many theories have been proposed. This study aimed to evaluate the occurrence of age-related changes affecting the chemosensory function (gustatory and olfactory) and general cognitive abilities and their potential associations in women. To this aim, 319 women (the age ranging from 18 to 92 years) were recruited and divided into four different age groups: 18–34 years, 35–49 years, 50–64 years, and ≥65 years. Our results confirmed that in women, gustatory, olfactory, and cognitive functions decline, though in a different manner during aging. Olfactory and cognitive function showed a slight decline along the first three age classes, with a dramatic decrease after age 65 years, while gustatory function decreased more gradually. Olfactory and gustatory deficits may have a high degree of predictivity for general cognitive function as well as for specific cognitive subdomains such as visuospatial/executive abilities, language, memory, and attention. Our study highlighted the importance of using chemosensory assessments for the early diagnosis of cognitive decline and for the development of appropriate personalized risk prevention strategies.

## 1. Introduction

Aging is a physiological, dynamic, and irreversible process that occurs throughout life, which inevitably involves all living beings. Human aging is defined as an end-of-life phase in which alterations occur in the biological, psychological, and social dimensions. During aging, the brain undertakes significant atrophy [1], which can be observed in the behavioral and functional changes in elderly subjects. The aging in the central nervous system (CNS) involves numerous changes, including a reduction in brain volume due to white and gray matter atrophy and the enlargement of the lateral ventricles [2]. Aging effects may also be found in sensory organs as responsible suppliers of environmental information to the CNS; so, a correct sensory processing is fundamental for physiological functions and psychological wellbeing. The effects of aging in sensory systems may affect the functionality of elderly subjects, making them even more vulnerable to disability and illness conditions as altered sensory processing leads to difficulties in modulating and organizing the intensity response, inducing hypo- or hypersensitivity to sensory inputs [3]. Sensory impairment may affect all sensory organs such as gustatory and olfactory ones, visual acuity, and hearing [4]. Olfactory and gustatory functions, compared to other sensory ones, have recently received increased attention due to the COVID-19 pandemic. In fact, numerous studies showed their relevance in contributing to individual psychophysical wellbeing [4,5]. Olfactory and gustatory pathways are strictly associated and may involve common brain areas such as the orbitofrontal cortex and amygdala [6]. Gustatory function plays an important role in the detection and identification of foods and beverages [7]. Gustatory deficits are classified as qualitative (dysgeusia and phantogeusia) and quantitative (ageusia, which is a total deficit, and hypogeusia or hypergeusia which are a decrease or increase in gustatory sensitivity, respectively). Gustatory impairments are usually associated with weight gain or weight loss and may lead to other health complications such as cardiovascular disease (such as high blood pressure) and diabetes. Gustatory dysfunctions affect around 5% of subjects and may be due to a decreased number of papillae in the mouth, changes in saliva composition, and impairment in brain areas [4]. The majority of gustatory deficits actually are linked to impairments in olfactory function as previously reported [8]. The decrease in gustatory and olfactory functions is a natural process during aging, which may have a negative impact on human life with decreased enjoyment in food intake, altered eating behavior, and poor nutrition.

Olfaction is a chemical sense that plays a fundamental role in human life to identify and avoid toxic substances and to regulate the correct food intake [2,9]. In fact, it has been shown that subjects with olfactory deficits are exposed to a greater risk of food poisoning and the inhalation of toxic gases [10,11]. Previous studies consistently reported that the occurrence of olfactory function impairment increases in relation to age [12,13]. Olfactory dysfunction is also associated with the regulation of emotional/motivational experiences, such as pleasantness and reward, anxiety, and social and reproductive behavior [14]. Moreover, previous studies showed that alterations in olfactory function are good predictors of neurodegenerative diseases, such as Parkinson’s and Alzheimer’s diseases [15,16,17,18], and that olfactory deficits are related to cognitive impairment, depression, apathy, and fatigue [19,20,21,22]. Olfactory impairment, both in physiological and pathological aging, may be influenced by a number of clinical and environmental factors other than age [13,23], such as sex/gender [24], cultural differences connected to the individual olfactory experience [25,26], infections [27], head trauma [28], and genetic factors [29].

Since previous studies [22,30] on both sexes indicated that gustatory and olfactory deficits were associated with an age-related decline in cognitive functions, our study focused, for the first time, on healthy women of different ages. Therefore, to delve deeper into the mechanisms of physiological aging in women, this study aimed at first to evaluate the presence of age-related changes affecting each taste modality (sweet, salty, sour, and bitter), each olfactory parameter (odor threshold, odor discrimination, and odor identification), each sub-score of cognitive abilities (visuospatial/executive, naming, memory, attention, language, abstraction, orientation), and then their potential association.

Furthermore, and in keeping of the potential role of chemosensory alterations as a prodromic factor of pathological outcomes such as dementia, Parkinson’s disease, Alzheimer’s disease, and others [8,31], additional regression analyses were also performed with the aim to develop predictive models for cognitive impairment.

## 2. Materials and Methods

### 2.1. Participants

In this study, 319 women (age range from 18 to 92 years) were recruited using convenience sampling from May 2019 to June 2023. Participants were divided into four different age groups: 18–34 years (n = 158), 35–49 years (n = 45), 50–64 years (n = 68), and ≥65 years (n = 48). Exclusion criteria were respiratory infections, such as chronic rhinitis or rhinosinusitis and asthma, neurodegenerative diseases, a history of head or neck trauma, stroke, diabetes, and any systemic disease associated with olfactory and gustatory disorders. Age, height (m), weight (kg), body mass index (BMI, kg/m^2^), smoking status, education, occupation, gustatory and olfactory function, cognitive abilities, and depression level were collected for all participants. The study was conducted in accordance with the Declaration of Helsinki and approved by the Local Ethics Committee (PROT. NP/2018/1630). All experimental procedures were explained to participants, who gave informed consent before the start of the experiment.

### 2.2. Procedures

The gustatory function was performed by the taste strips test (Burghart Messtechnik, Wedel, Germany). The taste strips test consists of filter paper strips impregnated with four concentrations of each basic taste quality: sweet, bitter, sour, and salty [32]. The taste strips test concentrations were the following: for a sweet taste (0.4, 0.2, 0.1, 0.05 g/mL of sucrose), for a bitter taste (0.006, 0.0024, 0.0009, 0.0004 g/mL of quinine hydrochloride), for a sour taste (0.3, 0.165, 0.09, 0.05 g/mL of citric acid), and for a salty taste (0.25, 0.1, 0.04, 0.016 g/mL of sodium chloride) [32]. Drinking water was used as a solvent in each taste modality and to rinse the participant’ mouth before the test. The total taste strips score may range from 0 to 16 and a taste score ≥ 9 is considered as normogeusia and score < 9 is classified as hypogeusia [32].

The olfactory function was assessed using the Sniffin’ Sticks test [33,34] which consists in three different sub-task odor thresholds (OTs), odor discrimination (OD), and odor identification (OI). First, the OT was detected using the n-butanol with 16 stepwise dilutions. The OT was assessed using a single-staircase technique based on three-alternative forced-choice (3AFC) tasks. Second, in the OD test, three pens were presented, two containing the same odor and the third containing the target odorant using 3AFC tasks. Third, OI was evaluated using 16 common odors, each presenting with four verbal descriptors in a multiple forced-choice format (three distractors and one target). Total scores (Threshold + Discrimination + Identification = TDI) were calculated. Scores ≤ 16, between 16.25 and 30.5, between 30.75 and 41.25, and >41.5 indicated functional anosmia, hyposmia, normosmia, and supersmellers [35].

For the evaluation of cognitive abilities, the Montreal Cognitive Assessment (MoCA) was used, which assesses cognitive impairment in different domains: visual–spatial skills, executive functions, attention and concentration, memory, language, conceptual thinking (abstraction), calculations, and spatial orientation [36,37]. The total score in the MoCA test is 30 and any score ≥ 26 is considered normal.

The depression level was evaluated using the self-reported Beck Depression Inventory (BDI-II) test [38], which includes 21 items with a four-point scale ranging in order of severity from 0 to 3. The depression level was classified as minimal, mild, moderate, and severe, for 0–13, 14–19, 20–28, and 29–63 scores, respectively.

### 2.3. Statistical Analyses

At first, a sample size calculation was performed in order to assess the required minimum number of subjects to be enrolled in the study. Based on previous studies using similar protocols [13,17,22,39], a number of about 300 total subjects can be considered adequate in order to detect differences in the variables investigated. In fact, a power calculation, performed considering a critical effect size of f = 0.20–0.25 (medium effect), with 90% power and a 5% significance level in a standard one-way ANOVA or a bivariate correlation, suggested a minimal required number of about 250 total subjects. Data were presented as mean values ± standard deviation (SD). Between subjects’ one-way ANOVAs and post hoc analyses using multiple pairwise comparison tests with Bonferroni’s corrected alpha values were carried out to assess statistical differences in gustatory (taste test), olfactory (Sniffin’ Sticks), and cognitive function (MoCA) and in other general parameters such as BMI, depression level (BDI), etc., in the four different age ranges (18–34, 35–49, 50–64, and ≥65 years). Bivariate correlations between gustatory, olfactory, and cognitive function were assessed using the Pearson’s coefficient (r^2^). These analyses were performed at first correlating all these factors also with the factor age, and then, because all of them correlated negatively with this factor, also controlling for it.

Furthermore, an exploratory stepwise multivariate linear regression analysis was performed in order to assess the potential contribution of each of the gustatory and olfactory parameters as predictors, measured as reported above, on the MoCA scores at each subscale (i.e., different cognitive subdomains) and on the total score as dependent variables.

Statistical analyses were performed using the SPSS software version 22 for Windows (IBM, Armonk, NY, USA). The significance level was set at *p* < 0.05.

## 3. Results

### 3.1. Sample Characteristics

The mean age of our total sample was 40.80 ± 19.36 years, with an age range of 18–92 years. As shown in Table 1, all the mean values related to the BMI, BDI, taste score, TDI score, and MoCA indicated that our sample falls within a normative range of healthy subjects.

### 3.2. Effect of Age on Gustatory, Olfactory, and Cognitive Function

**Figure 1:**
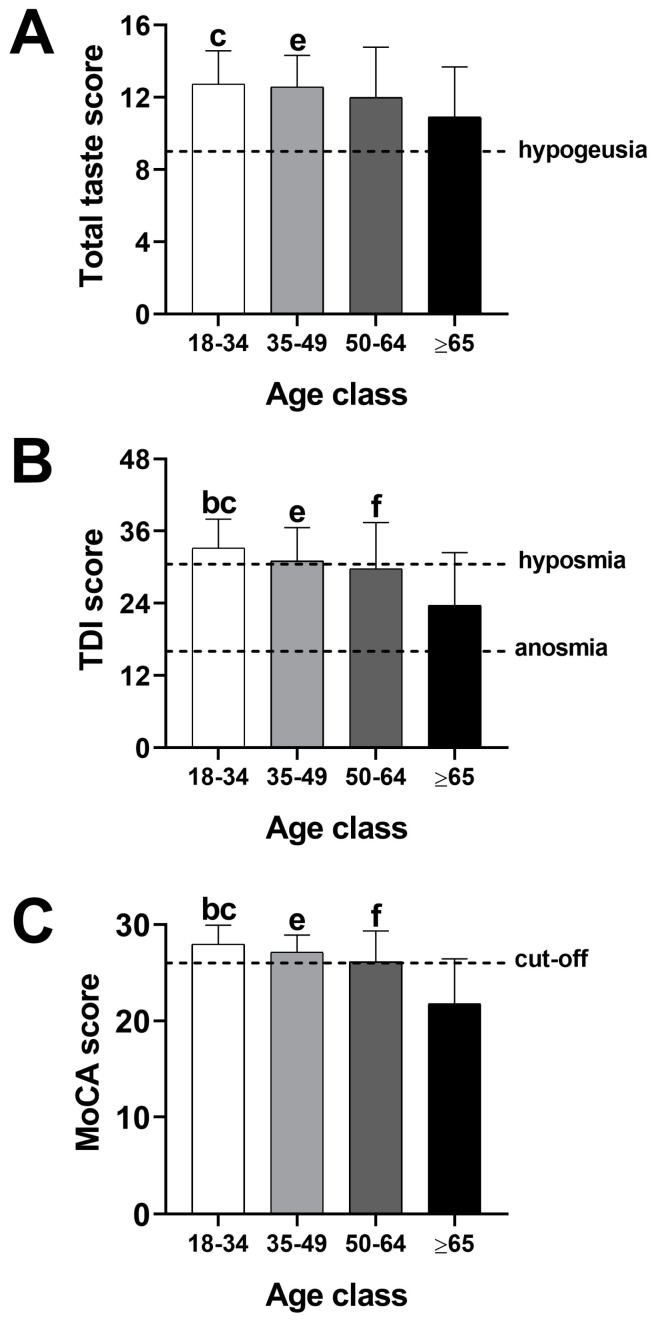
Mean values ± standard deviation of the mean (vertical bars) for the Total taste score (**A**), TDI score (**B**), and MoCA score (**C**) in different age classes (18–34, 35–49, 50–64, and ≥65 years). TDI = Total olfactory score; b = 18–34 vs. 50–64 years; c = 18–34 vs. ≥65 years; e = 35–49 vs. ≥65 years; f: 50–64 vs. ≥65 years (one-way ANOVA followed by Bonferroni’s post hoc test).

In order to assess the impact of age on gustatory, olfactory, and cognitive function, participants were divided into four age classes (18–34, 35–49, 50–64, and ≥65 years) and then compared by one-way ANOVA. The results of the general ANOVA are reported in Figure 1 and Table 2.

As shown in Figure 1, one-way ANOVA detected a significant age-related decline both in chemosensory (i.e., taste and olfaction) and cognitive function, as expected. Moreover, as a general rule, Bonferroni’s post hoc comparisons evidenced that the more robust differences were between the age class 18–35 and the age class ≥65 years. However, the three parameters were not affected by age in the same way. For instance, the TDI score as well as the MoCA score displayed a slight decline along the first three age classes and a dramatic decrease after age 65, while the Total taste decreased more gradually, and the differences were evident only between younger and older participants.

As shown in Table 2, similar differences in the age-related functional decline can be observed also in relation to the individual parameters related to each of the chemosensory and cognitive functions: for instance, in relation to gustatory function, Bitter continuously decreased, while salty displayed a fall in the ≥65 years age group. In relation to olfactory function, the OT constantly decreased along the four age classes, while the OD and OI decreased mostly in the ≥65 years age class. As regards the MoCA sub-scores, Orientation, Naming, and Visuospatial/executive abilities dramatically decreased in the ≥65 years age group, while Memory and Attention displayed a more gradual decrease with age. Interestingly, both Language and Abstraction displayed a strong decrease after age 50 (i.e., in the two older age classes). Among all the parameters considered, the only exception to this general trend was the Sweet taste that displayed a slight, though not significant, age-related decrease.

As reported in Table 3, further analyses also revealed significant differences in BMI scores among the four age classes but not in the level of depression as assessed by the BDI questionnaire.

Finally, significant differences were also observed in the distribution of women with or without a menstrual cycle along the four age classes (chi-square = 183.3, *p* < 0.0001). Accordingly, 12.7% (n = 20) of women in the age class 18–34 did not have a menstrual cycle, 57.8% (n = 26) in the age class 35–49, 91.2% (n = 62) in the age class 50–64, and 100% (n = 48) in the age class ≥ 65.

### 3.3. Correlations between Gustatory, Olfactory, and Cognitive Parameters

**Figure 2:**
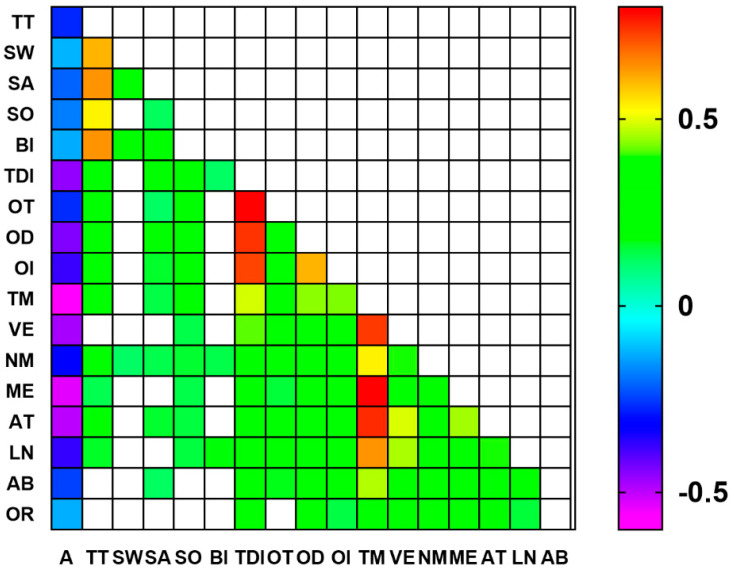
Correlation matrix between gustatory, olfactory, and cognitive parameters. Color codifications are reported only for statistically significant r^2^ Pearson values. Legend: A = Age; TT = Total taste score; SW = Sweet; SA = Salty; SO = Sour; BI = Bitter; TDI = Total olfactory score; OT = Odor threshold; OD = Odor discrimination; OI = Odor identification; TM = Total MoCA score; VE = Visuospatial/executive; NM = Naming; ME = Memory; AT = Attention; LN = Language; AB = Abstraction; OR = Orientation.

As shown in Figure 2, several correlations among gustatory, olfactory, and cognitive parameters emerged from our analyses. For instance, age negatively correlated with all the parameters considered, both the chemosensorial and cognitive ones. In particular, the TDI score and OD for the olfactory function and the total MoCA score, Visuospatial/executive, Memory, and Attention domains were highly correlated with age (i.e., Pearson’s r^2^ values higher than 0.4). Moreover, other correlations were detected between the three variable groups. For instance, Salty and Sour positively correlated will all olfactory parameters, Bitter correlated only with the TDI score, while Sweet was not correlated. However, when considering the Total taste, it significantly correlated with all the olfactory parameters and with the TDI score (r^2^ = 0.293). Sweet correlated with Naming and Salty with Naming, Attention, Abstraction and the MoCA total score. Sour correlated with Visuospatial/executive, Naming, Memory, Attention, Language, and the MoCA total score. Bitter correlated with Naming and Language and Total taste with Naming, Memory, Attention, Language, and the MoCA total score. All correlations were between r^2^ = 0.1 and 0.2, but that between Naming and Total taste was r^2^ = 0.229 and Naming significantly correlated with all the taste parameters. As regards the olfactory function, all MoCA domains resulted significantly correlated with all olfactory parameters (r^2^ values ranging from 0.144 to 0.414), and highly significant correlations were detected when considering the MoCA total score and the individual olfactory parameters OT, OD, and OI (r^2^ values ranging from 0.287 to 0.485).

**Figure 3:**
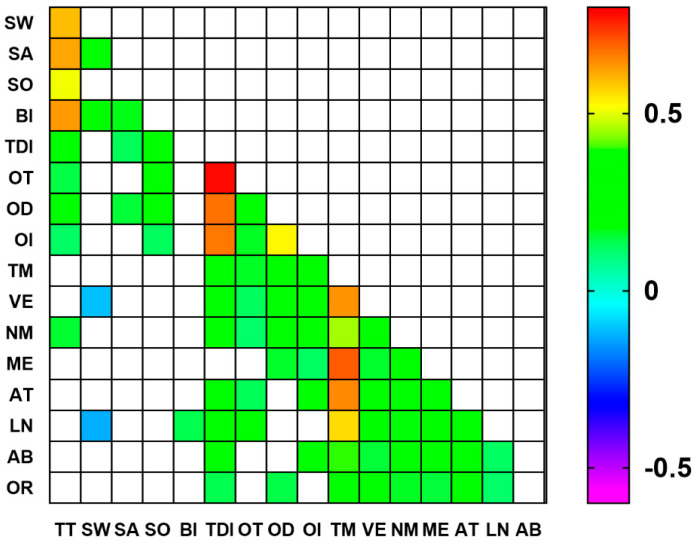
Correlation matrix between gustatory, olfactory, and cognitive parameters corrected for age. Color codifications are reported only for statistically significant r^2^ Pearson values. Legend: TT = Total taste score; SW = Sweet; SA = Salty; SO = Sour; BI = Bitter; TDI = Total olfactory score; OT = Odor threshold; OD = Odor discrimination; OI = Odor identification; TM = Total MoCA score; VE = Visuospatial/executive; NM = Naming; ME = Memory; AT = Attention; LN = Language; AB = Abstraction; OR = Orientation.

However, since age seems to have a prominent role as an intervening factor in the relationship between chemosensory and cognitive parameters, we aimed to investigate which of the above correlations was still present after controlling for the factor age. The results of this additional analysis are reported in Figure 3.

As shown in the Figure, after also controlling for the factor age, several significant correlations are still detectable. In particular, both Sour and Total taste score positively correlated with all olfactory parameters, while Sweet and Bitter did not. Salty positively correlated with the OD and TDI score. All correlations were between r^2^ = 0.1 and r^2^ = 0.2.

As regards the MoCA scores and gustatory function, the Visuospatial/executive domain negatively correlated with Sweet, while Language positively correlated with Bitter and negatively with Sweet. Naming positively correlated with the Total taste score.

Finally, as regards the MoCA scores and olfactory function, Visuospatial/executive and Naming subscales positively correlated with all parameters and with the TDI score, Memory correlated with OD and OI, Attention and Language with OT, while OI correlated with Attention, Language, Abstraction, and Orientation. Total MoCA score positively correlated with all the olfactory parameters and with the TDI score. All correlations were in the range between r^2^ = 0.114 and r^2^ = 0.298.

### 3.4. Multiple Regressions

Finally, based on the general hypothesis that alterations in chemosensory function could foster cognitive impairment (see Introduction), we carried out exploratory multiple regression analyses with the aim to investigate if these alterations could serve as potential predictive factors of deficits/decline in general cognition and/or specific cognitive subdomains.

Table 4 shows the models obtained by multiple regression for each of the MoCA subscales and for the MoCA total score using the individual gustatory and olfactory parameters as predictors.

As shown in Table 4, the three olfactory parameters predict the MoCA total score by about 25% and the Visuospatial/executive score by about 20%. Interestingly, the Language score is predicted by OT, OD, Bitter, and Sweet by about 15%, while the Attention score is predicted by about 15% by OI and OT. In contrast, Abstraction and Orientation seem to be less predictable using chemosensory parameters with values of variance explained lower than 10%. In general, olfactory parameters appear to be better predictors than gustatory ones for the general MoCA score and for almost all cognitive subscales.

## 4. Discussion

Olfactory and gustatory functions are emerging as potential biomarkers for cognitive impairment and neurodegenerative diseases [31]. Accordingly, previous studies [22,30] on both sexes indicated that chemosensory deficits were associated with an age-related decline in cognitive functions. The present study aimed, for the first time, to evaluate correlations between specific aspects of gustatory, olfactory, and cognitive abilities in healthy women of different ages. Our results reveal that in women, gustatory, olfactory, and all cognitive functions decline in relation to age, though in a different manner depending on the specific function or parameter considered. In particular, the olfactory and cognitive scores showed a slight decline along the first three age classes, with a dramatic decrease after age 65 years. Different theories have been proposed to explain the effect of aging on olfactory function such as the decreased number of fibers in the olfactory bulb due to brain atrophy, the reduction in the receptor cell regeneration, and the decrease in olfactory bulb volume [40]. The age-related decline in olfactory function occurred in a different manner, since the odor identification and odor discrimination remained constant up until the age of 65 years and then significantly decreased, while the odor threshold constantly decreased along the four age classes. The odor threshold is considered the level of an odor detection at low concentrations, meaning the least detectable concentrations of a smell that a subjects can perceive. Instead, odor identification and discrimination are the ability to indicate, discriminate, and identify a specific odor, respectively. These differences in the age-related decline for the olfactory parameters suggest that the odor threshold is more dependent on number of receptors expressed in the olfactory epithelium and on the periphery of the olfactory system [41], while odor identification and discrimination are more associated with the central brain areas such as the amygdala, the orbitofrontal, and the piriform cortex [42].

Thus, it is not surprising that the association between the decline in olfactory function and cognition observed in the present study appears to be stricter when considering olfactory discrimination and identification compared to the odor threshold. Similar results were already reported in previous studies from our group and other groups [43,44], pointing out a possible different involvement of cognitive abilities in these olfactory subcomponents.

Our results also display that gustatory function more gradually decreases, since gustatory impairment is less common in older adults, and it is strictly associated with olfactory deficits [4]. The most frequent causes of gustatory deficits are upper respiratory tract infections [28,40,45], altered saliva composition, a decreased number of papillae in the oral cavity, and brain atrophy. The precise mechanism of gustatory deficits in aging is not well known; however, many discriminants are associated with gustatory impairment such as oral infections and environmental and genetic factors. In addition, according to a previous study, our data also suggest that the perception of basic taste modalities was associated with age since the bitter taste perception constantly decreased in relation to aging, while salty taste displayed a fall in the ≥65 years [46]. A different age-related decrease in bitter taste gustatory perception has also been observed in other studies [47,48].

These age-related gustatory and olfactory impairments are usually considered part of the normal aging process [23,49]. Gustatory and olfactory impairments may lead to an inability in food flavor perception and may reduce, among others, eating enjoyment and reward. This decrease in eating enjoyment and reward may be at first associated with malnutrition and more in general to a lower quality of life and depression in patients.

In order to evaluate if chemosensory (i.e., gustatory and olfactory) dysfunctions may be considered an early sign to predict cognitive decline in a population of healthy women, we performed exploratory multiple regression analyses using the Total MoCA score and each of its subscales as a dependent variable. Our results suggest that olfactory dysfunction may predict cognitive decline as assessed by the Total MoCA score and in specific MoCA subscales as the Visuospatial/executive, Naming, Memory, and Attention ones. These results are consistent with previous studies indicating an association between olfactory function vs. Attention and Visuospatial/executive functions [50,51]. In particular, in our sample odor discrimination deficits were correlated with cognitive decline in Visuospatial/executive, Naming, and Memory abilities, while odor identification was associated with Attention, Visuospatial/Executive function, and Naming. Since olfactory deficits are associated with brain atrophy not only in the hippocampus but also in the entorhinal and orbitofrontal cortex [52], these results suggest, in line with previous studies, that an impairment in olfactory function may predict cognitive decline in older adults [53,54].

Moreover, the associations between olfactory dysfunction and impairment in executive and visuospatial abilities were reported also in patients with Parkinson’s disease [55]. Although other studies did not observe significant associations between olfactory parameters and Visuospatial/executive abilities [51,56], these discrepancies could be due to the different methodological approaches including both self-reported questionnaires and psychophysical clinical assessments for olfactory function and cognitive abilities. Among psychophysical clinical assessments for olfactory function, the University of Pennsylvania Smell Identification Test (UPSIT) examines only olfactory identification, while the Sniffin’ Sticks evaluates all three parameters of olfactory function as the threshold, discrimination, and identification, which further complicates direct comparisons among studies with these different methodologies. However, our data using the Sniffin’ Sticks test showed that Attention, Language, Visuospatial/Executive, and Memory were correlated with OI. Similarly, our previous study using Sniffin’ Sticks tests showed a significant correlation between OI and the MoCA total score [43]. In agreement with these findings, Li and colleagues [57] using the University of Pennsylvania Smell Identification Test (UPSIT) suggested correlations between OI vs. MoCA and mini-mental state examination total scores.

The clinical manifestation of cognitive impairment is a gradual pathological change in different brain areas over several years. The impairment in olfactory function for a long period of time in elderly subjects may reduce emotional memory (and reactivity) to external and environmental stimuli and may increase the risk of cognitive decline and dementia. The lack of adequate sensory stimulation could translate in missed or weakened perception, which in turn could negatively affect the neuroplastic processes in the brain during aging, thus promoting brain atrophy and therefore accelerating the neuropathological processes characteristic of dementia and other neurodegenerative pathologies or predisposing their development. This aspect might be also exacerbated in women due to the impacting changes in general physiology and brain functioning induced by the hormonal modifications caused by menopause. In fact, the hormonal changes underlying menopause, especially the drop in estrogen levels, may affect different aspects of the brain’s neurochemistry and plasticity, increasing the risk of developing cognitive impairments, dementia, and neurodegenerative disorders [58,59,60,61]. In this regard, it is not surprising that in our study, the most significant part of the decline in chemosensory and cognitive functions overlaps with the age in which premenopausal, menopausal, and postmenopausal conditions take place. Further studies involving precise hormonal evaluation could shed light on this important point through the investigation of a direct relationship between the changes in hormonal asset linked to menopause and the decline in chemosensory and cognitive functions observed in the present study along the different age classes.

Regardless, our results highlight the need to develop valid prevention tools to detect as early as possible potential risk factors and implement intervention strategies aimed on one hand at slowing down the degenerative processes and on the other to potentiate the residual abilities. In this regard, our multiple regression analyses emphasize the ability of a chemosensory assessment in providing a prediction on the general cognitive functioning (and also on specific subdomains such as Visuospatial/executive, Attention, Memory, and Language) in a population of healthy women. Yet, it has to be assessed, for instance through longitudinal and follow up studies, the possibility of using this kind of assessment in order to develop tools able to detect subtle or prodromic conditions associated with the later onset of degenerative conditions associated with cognitive impairment and decline.

At variance from what was observed for olfactory parameters, in our regression models, a significant relation between taste parameters and cognitive subdomains was found only for Language and sweet and bitter perception. Language is influenced by age, education, and demographic characteristics. This association between Language and sweet and bitter perception may be explained considering that the ability to perceive flavors occurs in the early stages of life with a natural preference for sweet foods, which contain high sources of energy and carbohydrates, while bitter taste is associated with aversive reactions and toxic foods. This implies a deep emotional characterization of these two flavors during development, involving brain areas deputed to the processing of the rewarding or aversive valence of stimuli such as the orbitofrontal cortex. Intriguingly, previous studies evidenced an involvement of the activation of this brain area in tasks where subjects were requested through verbal stimuli to imagine and/or think of specific flavors more than the brain areas directly involved in taste perception such as the insula and frontal operculum [62]. However, this hypothesis does not provide an explanation for the reasons why sweet taste is negatively while bitter taste is positively correlated with Language. Further studies are needed to better understand the nature and meaning of these relationships. In addition, a common pathway with the activation of the temporal lobe is reported for taste perception and language [63].

## 5. Conclusions

Our results show that chemosensory evaluations may have a high degree of predictivity for general cognitive functioning as assessed by the MoCA score as well as for specific cognitive subdomains such as Visuospatial/executive, Language and Naming, Memory, and Attention. Thus, it can be fruitfully utilized as a general tool for the early detection of risk conditions in order to prevent, or at least slow down, the course of physiological and/or pathological cognitive decline by applying programs of cognitive and/or chemosensory potentiation [64,65,66].

## Figures and Tables

**Table 1:** Descriptive statistics of the general sample.

Parameter	Mean	SD	95% C.I.
Body mass index	23.31	4.47	22.81–23.80
Beck Depression Inventory score	8.55	7.38	7.71–9.39
Total taste score	12.28	2.30	12.03–12.54
Sweet	3.40	0.78	3.31–3.48
Salty	3.31	0.94	3.20–3.41
Sour	2.52	1.01	2.41–2.63
Bitter	3.05	1.08	2.94–3.18
Total olfactory score	30.70	7.08	29.92–31.48
Odor threshold	6.44	4.29	5.97–6.92
Odor discrimination	11.56	2.48	11.28–11.83
Odor identification	12.54	2.54	12.26–12.82
Total MoCA score	26.52	3.50	26.13–26.91
Visuospatial/executive	4.56	1.02	4.45–4.67
Naming	2.91	0.36	2.87–2.95
Memory	3.15	1.59	2.98–3.33
Attention	5.46	0.95	5.35–5.56
Language	2.43	0.72	2.35–2.51
Abstraction	1.82	0.44	1.77–1.87
Orientation	5.94	0.37	5.90–5.98

Legend: SD = standard deviation; C.I. = confidence interval.

**Table 2:** One-way ANOVA and post hoc analyses performed on each of the parameters constituting the Total taste score, TDI score and Total MoCA score in the four age classes of the study.

	Age Group (Mean ± SD)				ANOVA		Post Hoc
	18–34	35–49	50–64	≥65	F	*p*	
TT	12.74 ± 1.84	12.58 ± 1.75	12.00 ± 2.78	10.91 ± 2.78	8.94	0.000	c; e
SW	3.47 ± 0.73	3.38 ± 0.68	3.40 ± 0.81	3.18 ± 0.94	1.62	0.184	---
SA	3.42 ± 0.79	3.47 ± 0.92	3.29 ± 1.00	2.79 ± 1.16	6.30	0.000	c; e; f
SO	2.71 ± 0.88	2.44 ± 1.16	2.32 ± 1.13	2.27 ± 1.00	3.84	0.010	b; c
BI	3.14 ± 1.04	3.29 ± 0.87	2.98 ± 1.01	2.67 ± 1.40	3.29	0.023	c; e
TDI	33.17 ± 4.79	31.02 ± 5.57	29.72 ± 7.69	23.64 ± 8.76	28.68	0.000	b; c; e; f
OT	7.46 ± 4.15	6.07 ± 3.85	5.90 ± 4.52	4.22 ± 3.86	8.27	0.000	c
OD	12.38 ± 1.72	11.69 ± 2.04	11.13 ± 2.83	9.33 ± 2.95	23.52	0.000	b; c; e; f
OI	13.12 ± 1.54	13.15 ± 2.15	12.54 ± 2.73	10.02 ± 3.57	23.54	0.000	c; e; f
TM	27.96 ± 1.98	27.13 ± 1.78	26.15 ± 3.19	21.77 ± 4.67	61.06	0.000	b; c; e; f
VE	4.83 ± 0.46	4.93 ± 0.25	4.60 ± 1.02	3.25 ± 1.63	45.64	0.000	c; e; f
NM	2.97 ± 0.16	2.96 ± 0.21	2.91 ± 0.28	2.65 ± 0.75	11.26	0.000	c; e; f
ME	3.87 ± 1.27	2.91 ± 1.33	2.81 ± 1.54	1.52 ± 1.46	38.97	0.000	a; b; c; e; f
AT	5.82 ± 0.46	5.49 ± 0.76	5.26 ± 0.96	4.52 ± 1.47	30.74	0.000	b; c; e; f
LN	2.60 ± 0.54	2.71 ± 0.46	2.34 ± 0.80	1.75 ± 0.86	24.37	0.000	b; c; d; e; f
AB	1.88 ± 0.36	1.93 ± 0.25	1.76 ± 0.49	1.58 ± 0.61	7.38	0.000	c; e
OR	5.95 ± 0.34	6.00 ± 0.00	6.00 ± 0.00	5.77 ± 0.69	4.63	0.003	c; e; f

Legend: TT = Total taste score; SW = Sweet; SA = Salty; SO = Sour; BI = Bitter; TDI = Total olfactory score; OT = Odor threshold; OD = Odor discrimination; OI = Odor identification; TM = Total MoCA score; VE = Visuospatial/Executive; NM = Naming; ME = Memory; AT = Attention; LN = Language; AB = Abstraction; OR = Orientation; a = *p* < 0.05 between 18–34 vs. 35–49 years; b = 18–34 vs. 50–64 years; c = 18–34 vs. ≥65 years; d = 35–49 vs. 50–64 years; e = 35–49 vs. ≥65 years; f: 50–64 vs. ≥65 years (one-way ANOVA followed by Bonferroni’s post hoc test).

**Table 3:** General parameters of the sample in relation to the four age classes.

	Age Group (Mean ± SD)				ANOVA		Post Hoc
	18–34	35–49	50–64	≥65	F	*p*	
BMI	22.18 ± 3.84	22.02 ± 2.85	25.00 ± 5.05	25.81 ± 5.09	14.45	0.000	b; c; d; e
BDI	8.67 ± 7.16	8.64 ± 6.94	7.15 ± 6.30	10.11 ± 9.92	1.27	0.283	---

Legend: BMI = body mass index; BDI = Beck Depression Inventory; b = 18–34 vs. 50–64 years; c = 18–34 vs. ≥65 years; d = 35–49 vs. 50–64 years; e = 35–49 vs. ≥65 years (one-way ANOVA followed by Bonferroni’s post hoc test).

**Table 4:** Multiple regression analyses with gustatory and olfactory parameters as predictors for MoCA total score and each of the MoCA subscales as dependent variables.

Predictors	B	SD Error	Beta	t	Significance (*p* Value)	F Value	*p* Value	R^2^
MoCA total score (dependent variable)								
OD	0.350	0.087	0.248	4.00	>0.000	36.58	0.000	0.258
OI	0.335	0.084	0.243	3.97	>0.000			
OT	0.128	0.042	0.157	3.08	>0.002			
Visuospatial/executive subscale (dependent variable)								
OD	0.097	0.027	0.234	3.63	0.000	25.31	0.000	0.194
OI	0.082	0.026	0.203	3.18	0.002			
OT	0.028	0.013	0.117	2.20	0.028			
Naming (dependent variable)								
OD	0.033	0.010	0.226	3.45	0.001	25.66	0.000	0.140
OI	0.027	0.009	0.191	2.92	0.004			
Memory (dependent variable)								
OD	0.229	0.034	0.365	6.78	0.000	46.02	0.000	0.127
Attention (dependent variable)								
OI	0.117	0.020	0.313	5.84	0.000	27.86	0.000	0.150
OT	0.036	0.012	0.164	3.05	0.002			
Language (dependent variable)								
OT	0.039	0.009	0.231	4.24	0.000	13.30	0.000	0.145
OD	0.048	0.016	0.165	3.02	0.003			
BI	0.113	0.036	0.171	3.15	0.002			
SW	−0.130	0.050	−0.141	−2.61	0.010			
Abstraction (dependent variable)								
OI	0.046	0.009	0.265	4.90	0.000	24.02	0.000	0.070
Orientation (dependent variable)								
OD	0.027	0.008	0.183	3.32	0.001	10.99	0.001	0.034

Legend: SW = Sweet; BI = Bitter; OT = Odor threshold; OD = Odor discrimination; OI = Odor identification.

## Data Availability

The datasets generated and analyzed during the current study are available from the corresponding author on reasonable request.
